# Contrast-Associated AKI in Hospitalized Adults With Sickle Cell Disease

**DOI:** 10.1016/j.ekir.2026.106600

**Published:** 2026-05-14

**Authors:** Loris Azoyan, Yannis Lombardi, Pablo Bartolucci, François Lionnet, Christelle Chantalat Auger, Sylvain Le Jeune, Louis Affo, Jean-Benoît Arlet, Jean-Philippe Haymann, Judith Leblanc, Olivier Steichen

**Affiliations:** 1 Réseau Sentinelles, Institut Pierre Louis d’Epidémiologie et de Santé Publique, INSERM, Sorbonne Université, Paris, France; 2 Service de Médecine Interne, Hôpital Tenon, Assistance Publique – Hôpitaux de Paris, Paris, France; 3 Drépanocytose: Groupe de Recherche de Paris (DREPS), GRC 25, Sorbonne Université, Paris, France; 4 Soins Intensifs Néphrologiques et Rein Aigu, Hôpital Tenon, Assistance-Publique - Hôpitaux de Paris, Paris, France; 5 Centre de référence de la drépanocytose et des maladies du globule rouge, Hôpital Henri Mondor, Assistance Publique - Hôpitaux de Paris, Créteil, France; 6 Service de Médecine Interne, Hôpital Kremlin-Bicêtre, Assitance Publique - Hôpitaux de Paris, Kremlin-Bicêtre, France; 7 Service de Médecine Interne, Hôpital Avicenne, Assistance Publique - Hôpitaux de Paris, Université Sorbonne Paris Nord, Bobigny, France; 8 Inserm U955, Institut Mondor de Recherche Biomédicale, INSERM, Paris, France; 9 Service de Médecine Interne, Hôpital Louis Mourier, Assistance Publique - Hôpitaux de Paris, Colombes, France; 10 Service de Médecine Interne, Hôpital Européen Georges Pompidou, Assistance Publique - Hôpitaux de Paris, Université Paris Cité, Paris, France; 11 Explorations fonctionnelles multidisciplinaires, Hôpital Tenon, Assistance Publique - Hôpitaux de Paris, Paris, France; 12 Institut Pierre Louis d’Epidémiologie et de Santé Publique, Sorbonne Université, INSERM, Paris, France; 13 Plateforme de recherche clinique Paris Est, Hôpital Saint Antoine, Assistance Publique - Hôpitaux de Paris, Paris, France

**Keywords:** acute kidney injury, contrast media, sickle cell disease

## Abstract

**Introduction:**

Patients with sickle cell disease (SCD) are at risk of chronic kidney disease (CKD) and acute kidney injury (AKI). The risk of AKI associated with contrast media (CM) exposures in this population is uncertain. The objective of this study was to investigate this temporal association in a multicentric case series.

**Methods:**

We performed a retrospective self-controlled case series (SCCS) in adults followed-up in SCD centers of the Greater Paris University Hospitals who experienced AKI during hospitalization between 2013 and 2022. SCCS estimates the relative incidence (RI) of events during exposure periods (around CM exposure) compared with control periods within the same individual. Analyses were restricted to hospitalizations periods and accounted for time-varying confounders such as age, presence of albuminuria, reduced estimated glomerular filtration rate, and stay in a medical ward or intensive care unit (ICU).

**Results:**

In the main analysis, that included 529 patients and 755 cases of AKI, AKI incidence increased significantly during the 7-day pre-exposure period (RI: 2.53, 95% confidence interval [CI]: 2.02–3.16), the 0 to 3 days postexposure (RI: 2.52, 95% CI: 1.99–3.19) and the 4 to 7 days postexposure (RI: 1.57, 95% CI: 1.20–2.06). Similar patterns were observed for stage 2 or 3 AKI, with higher RI.

**Conclusion:**

In this cohort of hospitalized adults with SCD, mainly young patients with preserved kidney function and few comorbidities, the RI of AKI was twice as high both before and after CM exposure, suggesting the role of underlying clinical conditions and related medical interventions rather than a causal effect of CM.

## Graphical abstract

**Figure:**
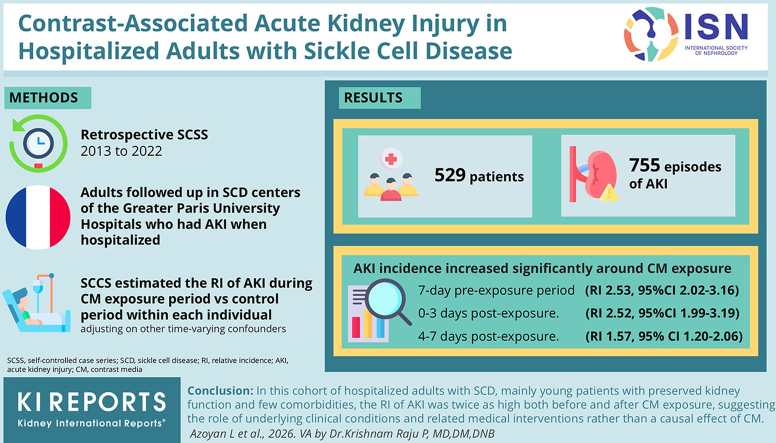

AKI is commonly defined by an abrupt decrease in kidney function occurring over 7 days or less.^1^ AKI is associated with increased short- and long-term mortality, as well as other long-term complications such as stroke, myocardial infarction, hypertension, and CKD.^2^^,^^3^ Contrast-associated AKI (CA-AKI) refers to AKI occurring within 24 to 72 hours of i.v. CM administration, without causal assumption.^4^, ^5^, ^6^ This definition followed several studies questioning the intrinsic role of CM and even its causal association with AKI.^7^, ^8^, ^9^ The latest evidence is reassuring regarding the risk of CA-AKI, it occurs in approximately 5% of patients who have an estimated glomerular filtration rate above 60 ml/min per 1.73 m^2^, and in up to 13% of patients regardless of their baseline kidney function.^4^^,^^10^ A recent meta-analysis of 21 studies found no significant difference in the overall risk of AKI between patients exposed to CM and propensity score-matched controls who underwent computed tomography without CM,^10^ but the risk could remain in specific clinical settings. For example, pre-existing CKD, notably an eGFR below 30 ml/min per 1.73 m^2^, intra-arterial CM administration, hypertension, and diabetes appeared as risk factors for the development of CA-AKI, suggesting that there may be a contrast induced component in this association.^10^, ^11^, ^12^

SCD, a genetic hemoglobinopathy, is 1 of the most prevalent monogenic diseases. In addition to distinctive acute events (e.g., vaso-occlusive episodes and acute chest syndromes), the natural history of SCD is marked by the development of chronic complications. Kidney damage is common— one third of adults with SCD have a rapid decline in renal function (≥ 3.0 ml/min per 1.73 m^2^ per year), and 11% evolve to end-stage renal disease.^13^, ^14^, ^15^ Patients with SCD frequently undergo radiological procedures involving the use of CM, in particular computed tomography pulmonary angiography which is performed in case of hospitalization for acute chest syndrome.^16^ These CM injections frequently occur with other confounding causes of AKI related to the clinical situation (e.g., worsened anemia, dehydration, ischemia-reperfusion kidney damage during vaso-occlusive episodes, sepsis, and hypoxemia during acute chest syndrome, etc.).^17^^,^^18^ Only 1 study investigated the risk of CA-AKI among patients with SCD.^19^ This monocentric retrospective series of 79 patients showed that transient AKI occurred in 1.6% of patients within 72 hours after CM injection, but it did not investigate risk factors, such as the care settings in which CM injection was performed (ambulatory care, hospitalization in a medical ward, or hospitalization in an ICU), or baseline renal function.

In this multicenter study, we investigated the temporal association between CM administration and AKI in patients hospitalized with SCD, taking into account the settings of CM administration and other confounding factors.

## Methods

### Study Design

We conducted an observational multicenter retrospective SCCS study using the clinical data warehouse of Greater Paris University Hospitals (Assistance Publique – Hôpitaux de Paris). Data collected during routine care in 38 hospitals in Paris and the Ile-de-France area are automatically included in the clinical data warehouse. The research database follows the Informatics for Integrating Biology and the Bedside standard.^20^

The study was approved by the Assistance Publique – Hôpitaux de Paris clinical data warehouse scientific and ethics committee (IRB00011591, CSE-EDS n°22-02). Final data extraction was performed on March 9, 2022 using the Integrating Biology and the Bedside platform. No linkage was made with other databases. The manuscript was written according to the reporting of studies conducted using observational routinely collected health data statement (Supplementary Table S1).^21^

### Inclusion Criteria

Potentially eligible patients consisted of all adult patients with an International Classification of Diseases (ICD)-10 code for SCD (D570, D571, D572, D578) recorded in 1 of the 5 Assistance Publique – Hôpitaux de Paris adult SCD center of Avicenne, Henri Mondor, Kremlin Bicêtre, Louis Mourier, or Tenon hospital. Patients also had to be ≥ 18 years, have consulted or been hospitalized at least once after August 8, 2017 (ensuring individual information on clinical data reuse for research purposes noted in their medical reports), and with a minimum of 2 creatinine measurements spanning over at least 7 days. Additionally, patients included in the analysis had to have experienced at least 1 AKI (regardless of their exposure to CM) and a confirmed SCD based on medical reports. Patients with a stable eGFR < 15 ml/min per 1.73 m^2^ , those requiring long-term dialysis, or those who had undergone renal transplantation at the start of follow-up were not included.

### Data Collection and Identification of Genotypes

For the included patients, the following Assistance Publique – Hôpitaux de Paris data were collected from September 1, 2013 to March 9, 2022: demographic data, dates of hospital visits (emergency department, medical wards, and ICU), medical reports, ICD-10 codes with the corresponding visit, procedures codes, and biological values with dates, including creatinine measurements and urinary protein assessments. Comorbidities were identified if the corresponding ICD-10 code was recorded at least once before occurrence of the first AKI episode. Main reason for hospitalization was identified at each visit.

The use of regular expressions (regex), that is, a sequence of characters that specifies a match pattern in text, enabled to confirm SCD from free text medical reports. For each patient, the number of occurrences of each genotype (SS, SC, Sβ^0^, Sβ^+^) was counted across all medical reports. If more than 1 genotype was found for a patient, the reports were then checked manually by an expert physician (LA). Patients with a medical history consistent with the disease (in particular admission for vaso-occlusive episodes) but whose genotype could not be determined were included as unclassified.

### SCCS Method

The SCCS method was initially developed to investigate the temporal association between vaccine and suspected adverse effects using only cases information.^22^ In this study, it enables to estimate the RI of AKI events during exposure periods (1 week after CM administration) and pre-exposure periods (1 week before CM administration), compared with control periods (any period outside of the week around CM exposure) within the same individual.^23^ Of note, SCCS does not allow estimates of absolute incidence, only estimates of RI.^24^ The AKI occurrence was modelled as a nonhomogeneous Poisson process conditioned on the occurrence of the AKI event and the individual’s exposure history to CM and other time varying confounders detailed below. Consequently, only individuals who experienced AKI were included in the analysis. Although subjects unexposed to CM do not contribute to the estimation of the impact of CM exposure, they contribute to the estimation of other effects, such as age, and are therefore kept in the analyses.^25^ One of the main advantages of the SCCS model is that RI parameters are estimated within subjects meaning that time-invariant confounders, known or unknown, are automatically accounted for. For example, the *APOL1* gene variants G1 and G2 are frequent in subjects from African descent and associated with SCD nephropathy but rarely screened.^26^^,^^27^ The model also allows adjustment for time-varying exposures, which in the standard SCCS model used here, are specified during time intervals, requiring continuous variables such as age and kidney function to be modeled using discrete categories.

**Figure 1:**
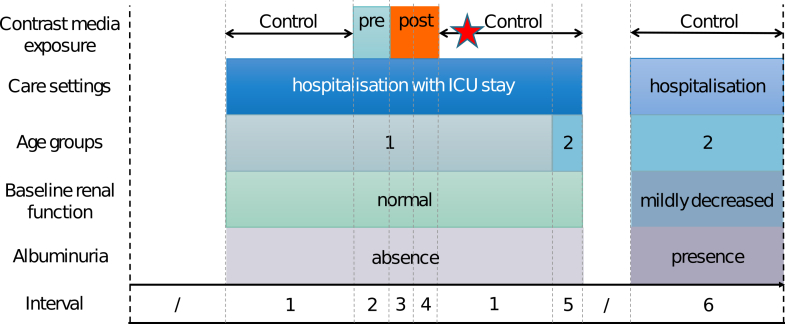
SCCS design for the main analysis using the timeline of a hypothetical patient evolving over time. The observation periods, restricted to hospitalizations, are segmented into intervals depending on the time from CM exposure and the combinations of time-varying confounding factors. AKI is represented by the red star. AKI, acute kidney injury; CM, contrast media; ICU, intensive care unit; SCCS, self-controlled case series.

Two analyses were performed. The main analysis included only hospitalization periods, with exposures periods truncated accordingly, as creatinine measurements (needed for AKI event ascertainment) are routinely performed during hospital stays and therefore less dependent on CM exposition. Analysis was adjusted for age group, CM exposure, and pre-exposure, change in baseline GFR (matching the Kidney Disease: Improving Global Outcomes [KDIGO] CKD stages), presence of albuminuria, hospitalization with intensive care stay, and a terminal risk period (Figure 1). The second analysis (full follow-up analysis) was not restricted to hospitalization period. It was adjusted for the same time varying exposures but 3 adjustment variables for settings of care were used as follows: emergency visit without subsequent hospitalization, hospitalization without intensive care stay, and hospitalization with intensive care stay. Ambulatory care was considered the baseline risk.

### Study Periods and Exposure Periods

The study observation period started for each patient on the date of the first available creatinine measurement at age ≥ 18 and ended on the date of the last available creatinine measurement. The observation period was censored when patients had a stable eGFR <15 ml/min per 1.73 m^2^, death, or required long-term dialysis or renal transplantation.^28^

The CM exposure period corresponded to the 7 days following the injection the date of CM injection based on procedure codes. This period was subdivided from day 0 to day 3 and from day 4 to day 7. The day 0 to 3 period reflects the conventional definition of CA-AKI.^9^ In line with KDIGO guidelines indicating that AKI can occur up to 5 days after CM exposure, the window was extended to 7 days to match the general KDIGO AKI definition and previous studies using extended observation periods.^1^^,^^29^^,^^30^ A pre-exposure period of 7 days was also defined (details below). The control periods, that correspond to the baseline risk, are any periods of hospitalization time outside the 7 days pre-exposure and 7 days postexposure to CM.

### Adjustment of Time Varying Confounders

Age is an established risk factor for AKI.^31^ We used 20 age groups separated by quantiles according to the age at first event. The effect of age was assumed to be constant within each interval. Increasing the number of age categories up to 40 did not improve model fit, so 20 groups were retained to avoid unnecessary complexity.

We used the settings of care as a proxy to reflect the clinical status of patients. We collected the start and end dates of emergency department visits without subsequent hospitalization and hospitalizations with or without intensive care stay. In the main analysis, restricted to hospitalization, baseline risk was hospitalization without intensive care stay. In the full follow-up analysis, baseline risk corresponds to outpatient periods including consultations and day-care hospitalizations.

We used the 2009 CKD-Epidemiology Collaboration equation without adjusting for ethnicity for estimating eGFR, as it most closely approximates measured GFR in patients with SCD.^32^ An eGFR greater than 90 ml/min per 1.73 m^2^ (with or without hyperfiltration) was considered the baseline risk. Reduced eGFR stages were defined per KDIGO as follows: 60 to 89 (mildly decreased), 45 to 59 (mild to moderate), 30 to 44 (moderate to severe), 15 to 29 (severe), and <15 ml/min per 1.73 m^2^ (kidney failure). To estimate reduced eGFR stages, we used creatinine measurements made in the outpatient setting, and considered dates from which stage changes were persistent over 6 weeks. To establish the period associated with each reduced eGFR stage, we considered that once a patient was in a specific stage, they could not return to a milder stage but only evolve to a later stage.

Albuminuria was defined using a threshold of 300 mg/g for albumin-to-creatinine ratio and 500 mg/g for total protein-to-creatinine ratio. Only measurements taken in outpatient settings were used, with a minimum interval of 30 days between consecutive measurements. Presence of albuminuria was confirmed with at least 2 out of 3 consecutive measurements above the threshold. The albuminuria exposure period started at the date of the first above-threshold measurement and was considered permanent for the remainder of the observation period.

A 30-day terminal risk period was included to account for the increased likelihood of follow-up ending after an AKI event (e.g., because of initiation of long-term dialysis, or death). Baseline risk corresponds to the observation period excluding these 30 days (details below in model assumptions).

### Primary Outcome

The primary outcome is the incidence of AKI according to the KDIGO definition based on creatinine changes in a maximum of 7 days.^1^ Urine-output criteria were not used, as they are inconsistently recorded outside ICUs. We used a conservative definition of AKI to minimize potential misclassification that could occur using electronic health databases. This approach was deemed appropriate given the frequency of creatinine measurements during hospital stays. Stage 1 AKI was defined as an increase in serum creatinine ≥ 0.3 mg/dl (≥ 26.5 μmol/l) within 2 days or 1.5 to 1.9-fold within 7 days; stage 2 as 2 to 2.9-fold increase; and stage 3 as ≥ 3-fold or ≥ 4.0 mg/dl (≥353.6 μmol/l) within 7 days. The date of the outcome was the day of the first creatinine increase matching these criteria. Events separated by 7 days or more were considered as different events. Results are presented for all AKI, stage 2 or 3, and stage 3 alone, to account for the temporal dynamics of creatinine rise and reduce misclassification because of variability in blood sampling timing. In a sensitivity analysis, we applied a modified KDIGO definition adapted for children with SCD (sKDIGO), which excludes a 1.5-fold creatinine increase from 0.2 mg/dL (17.7 µmol/L) or less, to 0.3 mg/dL (26.5 µmol/L), from the AKI definition, all other criteria remaining unchanged.^33^

### Model Assumptions

The SCCS model assumes the following 4 hypotheses: (i) AKI events are uncommon or follow a Poisson distribution, (ii) AKI events do not influence the length of observation periods, (iii) AKI events do not influence subsequent exposures to CM, and (iv) CM exposures do not influence AKI event ascertainment. To evaluate the robustness of the model and to mitigate the potential consequences of not meeting assumptions (i) to (iii), we applied strategies recommended by the original authors of the SCCS method.^34^

Assumption (i) was assessed with histograms of gap times between consecutive AKI episodes, and a sensitivity analysis including only first events was conducted.^24^

Assumption (ii) was investigated using histograms of the time interval between events and at the end of the observation period. A 30-day terminal risk period was then included in each analysis to account for the increased likelihood of follow-up ending.^35^ The 30-day duration was selected based on graphical inspection and clinical rationale. Sensitivity analysis without the terminal risk period were performed.

Assumption (iii), that events do not influence subsequent exposures, must be met to avoid bias in the estimated RI. This assumption is not met in our study. First, a computed tomography scan with contrast may be ordered in response to a clinical situation that may cause AKI before or after CM injection. Second, clinicians may choose to delay CM administration following an episode of AKI. We included a pre-exposure risk period consistent with methodological recommendations for handling event-dependent exposure delays.^34^, ^35^, ^36^ This approach was preferred over alternative SCCS models for event-dependent exposures, which do not allow a pre-exposure period.^37^ This period was defined as the 7 days before CM exposure, and the influence of its duration on RI estimates was evaluated by varying the pre-exposure period from 0 to 21 days within the same hospitalization.

Assumption (iv) was investigated graphically to evaluate the creatinine measurement before and after exposure to CM.

### Statistical Analysis

All analyses used R (version 4.0.0, R Foundation, Vienna, Austria) software.^38^ SCCS was fitted using the SCCS package.^34^^,^^39^ Estimates are presented with RI and 95% confidence intervals (CIs). A 2-sided *P*-value of < 0.05 was considered statistically significant.

## Results

### Study Population

**Figure 2:**
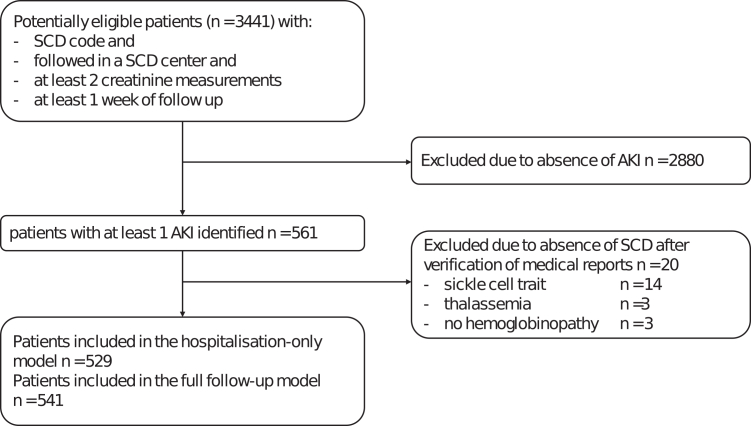
Flowchart of patients included in both the main analysis and the full follow-up analysis. AKI, acute kidney injury; SCD, sickle cell disease.

Among the 3411 potentially eligible adult patients with SCD, 529 (15.5%) experienced at least 1 AKI during hospitalization and were included in the main analysis (Figure 2). They experienced 755 AKI in total. Patient characteristics are summarized in Table 1. Median age at inclusion was 29.7 years and the most frequent genotype was homozygous SCD (SS, 86.2%). The most common comorbidities before the first AKI event were acute chest syndrome/pneumonia (both conditions are grouped together as no specific ICD-10 code exists for acute chest syndrome), heart failure, and hypertension. At baseline, the majority of patients had preserved kidney function; 95 (18.0%) had an eGFR below 90 ml/min per 1.73 m^2^. At the end of the observation period, 143 (27.0%) had an eGFR below 90 ml/min per 1.73 m^2^, including 22 (4.2%) between 45 to 59 ml/min per .73 m^2^, 27 (5.1%) between 30 to 44 ml/min per 1.73 m^2^, and 34 (6.4%) between 15 to 29 ml/min per 1.73 m^2^. Among the 529 included patients, 88 had no outpatient urinary protein measurement available, and 85 had only 1 or 2 measurements, of whom 29 (34.1%) had at least 1 value above threshold. Among the 356 patients with sufficient measurements, 132 (37.1%) had confirmed albuminuria, representing 25% of the full cohort, including 52 (14.6%) identified within the first year of observation. Patients with insufficient measurements were considered unexposed in the SCCS analyses.

**Table 1:** Patient characteristics in the main analysis

Variables	Study population for the main analysis
Number of patients	529
Median age at inclusion	29.7 (21.9–41.0)
Women	295 (55.8%)
Genotype	
SS	449 (86.2%)
SC	45 (8.5%)
Sβ+	11 (2.1%)
Sβ0	11 (2.1%)
Unclassified	13 (2.5%)
Comorbidities before first AKI	
Acute chest syndrome/pneumonia^a^	232 (43.9%)
Hypertension	97 (18.3%)
Heart failure	113 (21.4%)
Ischemic heart disease	24 (4.5%)
Rhythms and conductions disorders	49 (9.3%)
Pulmonary embolism	66 (12.5%)
Pulmonary arterial hypertension	40 (7.6%)
Cerebrovascular accident	35 (6.6%)
Diabetes mellitus	17 (3.2%)
Malignancy	27 (5.1%)
Median observation length (days)	73 (37–115)
Total observation length (person-years)	188.73
Baseline eGFR	
≥ 90 ml/min per 1.73 m^2^	434 (80.2%)
60–89 ml/min per 1.73 m^2^	50 (9.2%)
45–59 ml/min per 1.73 m^2^	13 (2.4%)
30–44 ml/min per 1.73 m^2^	21 (3.9%)
15–29 ml/min per 1.73 m^2^	11 (2.0%)
Patients with ≥ 3 outpatient urinary protein measurements	356 (67.3%)
Albuminuria identified in the first year of follow up	52 (14.6%)
Number of creatinine measurement	
Total	27,721
By patient	36 (18–69.5)
Number of outpatient urinary protein measurement	
Total	3517
By patient	7 (3–11)
Number of CM exposure	
Total	1669
By patient	2 (1–5)
Age at first CM exposure	30.4 (23.9–40.7)
Unexposed patients during the period of interest	84 (15.9%)
Total number of imaging procedures with CM	1850
CT pulmonary angiogram	907 (49.0 %)
Abdomen and pelvis CT	266 (14.4 %)
CT of 3 or more regions	232 (12.5 %)
Chest CT	108 (5.8%)
Head CT	83 (4.5%)
Others	234 (12.6%)
Number of hospitalizations without intensive care stay	
Total	8006
By patient	8 (3–17)
Number of hospitalizations with intensive care stay	
Total	756
By patient	0 (0–1)
Number of AKI	
Total	755
By patient	1 (1–2)
Age at first AKI	33.4 (25.3–44.6)
Number of stage 2 or 3 AKI	
Total	169
By patient	0 (0–1)
Number of stage 3 AKI	
Total	65
By patient	0 (0–0)

AKI, acute kidney injury; CM, contrast media; CT, computed tomography; eGFR, estimated glomerular filtration rate.

The total number of imaging procedures exceeds the total number of CM exposures because some patients underwent multiple imaging at the same time. Categorical variables are presented with counts and percentages, numerical variables with medians, first, and third quartile.

a Acute chest syndrome and pneumonia are grouped together as no specific ICD-10 code exists for acute chest syndrome. Acute chest syndrome is often coded as pneumonia and pneumonia also fulfills its diagnostic criteria.

Among the 8762 hospitalizations, vaso-occlusive crisis was the main reason for admission in 6462 stays (73.8%). A total of 1318 stays (15.0%) had at least 1 infectious code recorded, with pneumonia or acute chest syndrome being the most frequent (761 stays, 8.7%). Stays with AKI were significantly longer than those without (median 12 days [IQR 7–22.5] vs. 4 days [IQR 1–7], Wilcoxon rank-sum test *P* < 0.001). However, AKI occurred at a median of day 4 (IQR 1–8) within the hospitalization. Median observation length (cumulated hospitalization) was 73 days, resulting in an overall cohort follow-up of 188.7 person-years. There were 169 AKI of stage 2 or 3 and 65 AKI of stage 3. Median number of CM exposure was 2 (IQR: 1–5), 84 patients were not exposed to CM during hospitalization. The event counts and total durations of the different exposure periods are summarized in Table 2.

**Table 2:** Counts of events and total duration of exposure periods in person-years for the main analysis

Exposure	Number of AKI	Person-years
Total	755	188.73
CM exposure		
7 d pre-exposure	132	14.34
0–3 d post exposure	104	11.58
4–7 d post exposure	73	13.11
Outside 7 d pre- or postexposure	446	149.70
Settings of care		
Hospitalization	564	151.88
Hospitalization with intensive care stay	191	36.85

AKI, acute kidney injury; CM, contrast media.

### Relative Incidences

**Figure 3:**
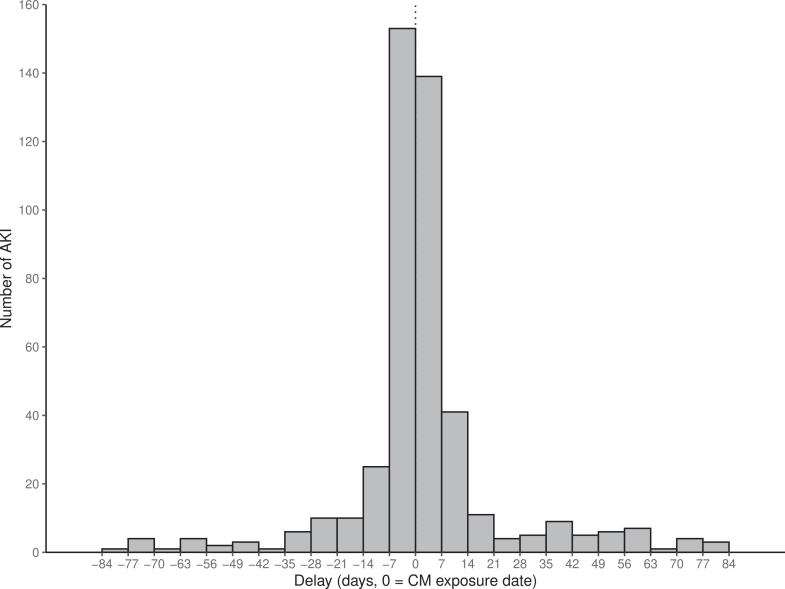
Histogram of the time between AKI and the closest CM exposure in the main analysis. As patients could have multiple AKI events and multiple CM exposures, the shortest absolute time difference with any CM exposure was used for each AKI event. CM, contrast media. AKI, acute kidney injury; CM, contrast media.

RIs of AKI for CM exposures and pre-exposure, hospitalization with ICU stay, and terminal risk period obtained in the main analyses are reported in Table 3. The delay between AKI and the closest CM exposure is represented in the histogram in Figure 3. The RIs of AKI were 2.02 (95% CI: 1.65–2.47, *P* < 0.001) for the 0 to 7 days post CM exposure and 2.52 (95% CI: 2.01–3.15, *P* < 0.001) for the whole 7 days pre-CM exposure. A similar pattern was observed for stage 2 or 3 AKI. Age effect for all AKI is represented in Supplementary Figure S1. RI estimates for hospitalization with ICU stay may not always fully meet all SCCS assumptions, as the model was primarily designed to assess the effect of CM exposure, and should be interpreted with caution. Similarly, RIs of various reduced eGFR stages and presence of albuminuria might not be reliable and are not shown.

**Table 3:** Relative incidence of acute kidney injury in the main analysis (529 patients)

Exposure	Relative incidence	95% CI	*P* value
Acute kidney injury (*n* = 755)			
7 d pre-CM exposure^a^	2.53	2.02–3.16	< 0.001
0–3 d post-CM exposure^a^	2.52	1.99–3.19	< 0.001
4–7 d post-CM exposure^a^	1.57	1.20–2.06	< 0.001
Hospitalization with intensive care stay^b^	1.66	1.29–2.14	< 0.001
Terminal-risk period^c^	1.79	1.20–2.66	0.004
Stage 2 or 3 AKI (*n* = 169)			
7 d pre-CM exposure^a^	4.09	2.56–6.52	< 0.001
0–3 d post-CM exposure^a^	3.65	2.19–6.08	< 0.001
4–7 d post-CM exposure^a^	3.33	2.01–5.53	< 0.001
Hospitalization with intensive care stay^b^	1.41	0.80–2.48	0.19
Terminal-risk period^c^	3.42	1.59–7.34	0.001
Stage 3 AKI (*n* = 65)			
7 d pre-CM exposure^a^	6.12	3.01–12.62	< 0.001
0–3 d post-CM exposure^a^	5.32	2.40–11.77	< 0.001
4–7 d post-CM exposure^a^	3.46	1.45–8.26	0.005
Hospitalization with intensive care stay^b^	2.37	0.76–7.40	0.14
Terminal-risk period^c^	2.87	0.89–9.24	0.08

AKI, acute kidney injury; CI, confidence interval; CM, contrast media.

Analyses accounts for pre- and post-CM exposure, hospitalization in ICU, reduced baseline eGFR stages, age, and a terminal-risk period of 30 days. Analyses were not adjusted on baseline eGFR for stage 3 AKI given the lower number of events.

a Reference is the period outside the 7 days before and 7 days after CM exposure for the same patient.

b Reference is hospitalization without intensive care stay.

c Reference corresponds to the remainder of the observation period, excluding these 30 days.

### Sensitivity Analyses and Verification of the Model Assumptions

In the full follow-up analysis, 541 patients were included and experienced 881 AKI in total (Figure 2). Their characteristics are presented in Supplementary Table S2, the event count and durations in Supplementary Table S3. RIs were consistent in the full follow-up analysis, with a RI of AKI of 3.11 (95% CI: 2.48–3.90, *P* < 0.001) for the 0 to 3 days post-CM exposure, 2.00 (95% CI: 1.55–2.58, *P* < 0.001) for the 4 to 7 days post CM exposure, and 2.90 (95% CI: 2.34–3.60, *P* < 0.001) for the 7 days pre-CM exposure (Supplementary Table S4).

Histogram of the gap times between AKI events showed a mode close to zero suggesting there may be clustering of events (assumption i, Supplementary Figure S2). Analyses restricted to first AKI event showed consistent results with analysis including all AKI events (Supplementary Table S5). In the main analyses, the RIs of AKI restricted to first event only were 2.75 (95% CI: 2.04–3.71, *P* < 0.001) for the 0 to 3 days post-CM exposure, 1.65 (95% CI: 1.17–2.32, *P* < 0.001) for the 4 to 7 days post-CM exposure, and 2.73 (95% CI: 2.04–3.64, *P* < 0.001) for the 7 days pre-CM exposure. 21 AKI events (2.78%) were excluded after applying the modified KDIGO definition for children with SCD, whereas the RIs remained similar (Supplementary Table S5).

Histogram of gap time between AKI and the end of observation showed a mode close to zero indicating that AKI may be followed by the end of the observation periods, hence the use of a 30-day terminal risk period (assumption ii, Supplementary Figure S3). Analyses were repeated without the terminal risk periods and showed similar RI during pre- and postexposure periods (Supplementary Table S5).

Across all analyses, the RI of AKI during the pre-exposure period was in the same order of magnitude as in the post exposure ones. The impact of the pre-exposure length on the RI of both the pre-exposure period and 0 to 3 days post-CM exposure is represented in Supplementary Figure S4 (assumption iii). The postexposure RIs remained stable with longer pre-exposure durations during the same hospitalization. The analysis was also repeated by partitioning the pre-exposure period into 2 periods of 7 to 4 days and 3 to 0 days pre-exposure to CM and showed consistent results (Supplementary Table S5).

The delay between CM exposure and creatinine measurements appears globally symmetrical in the main analysis, suggesting that exposure does not influence event ascertainment (assumption iv, Supplementary Figure S5).

## Discussion

### Key Results

In our cohort, AKI affected about 15% of patients during hospitalization, highlighting the renal vulnerability of adults with SCD. The SCCS analysis showed that the RI of AKI during hospital stays is roughly 2 times greater during the week after CM exposure than at baseline, and even higher for stage 2 or 3 AKI. However, the RI of AKI in the week before CM exposure is equally high.

### Interpretation

The focus on the postexposure risk in previous studies does not capture all potential temporal dynamics between AKI and CM exposure and may explain the overestimation of the risk of CM-induced AKI.^29^ Our results are consistent with more recent studies that used strategies such as a propensity score to better control for indication bias and those that did not show an increased risk of AKI after CM exposure.^9^^,^^10^

Indeed, the similar AKI risk observed before and after CM exposure in our hospitalized patients suggests that CM had no causal role in the development of these AKI but more probably that an underlying acute health condition both required a radiological procedure with CM injection and was at risk of AKI. These results are consistent with current guidelines that advocate contrast-enhanced imaging whenever clearly indicated, even in vulnerable patients.^4^, ^5^, ^6^

### Strengths and Limitation

This multicenter study included a large number of patients with confirmed SCD. The SCCS design was chosen as it is suited to investigating the temporal association between AKI and CM exposure, providing a complementary perspective to more commonly used approaches such as propensity-matched cohort designs.^10^ In addition, SCCS inherently controls for all time-invariant confounders, and analyses were further adjusted for some key time-varying confounders, including age, hospitalization with intensive care stay, reduced baseline eGFR, and presence of albuminuria. AKI were defined on the basis of changes in creatinine measurement according to the KDIGO guidelines and not on the basis of ICD-10 code, which underestimates their incidence.^40^, ^41^, ^42^ Results were consistent across the various sensitivity analyses.

The main limitations of our study are inherent to the use of routinely collected health data. Selection bias was addressed by defining SCD as a compatible ICD-10 codes, follow-up in a reference center, and confirmation from free text in medical reports. Exposure to CM was identified with French procedure codes. Specific validation studies for their accuracy are lacking. However, they are used for reimbursement by the national health insurance service and subject to administrative quality controls. Despite the advantages of the biological definition of AKI based on creatinine measurements, more subtle, subthreshold creatinine changes, may have been missed. Short term fluctuations in hydration status and muscle mass could also affect creatinine concentration and lead to misclassification. However, these factors are not specific to SCD and are unlikely to explain the consistent findings for stage 2 or 3 AKI.

Potential confounding medications, such as angiotensin-converting enzyme inhibitors and nonsteroidal anti-inflammatory drugs, could not be taken into account because dispensing data were not available in the clinical data warehouse. Illness severity was approximated by ICU stay, but the various types of acute conditions, notably infectious diseases, could not be explicitly defined within the SCCS model. Chronic conditions such as diabetes, hypertension, or heart failure were not included, as their timing cannot be reliably determined in retrospective data. However, these conditions are generally stable over short periods of time limiting their potential confounding effect. The inclusion of age as a time-varying covariate partially accounts for changes in baseline risk. Reduced eGFR and albuminuria were incorporated as time-varying covariates to improve the precision of CM exposure estimates; however, they may not satisfy SCCS validity assumptions, and their RI estimates are not reported, as the study was not designed to assess their effect on AKI risk. Baseline eGFR categories grouped values ≥ 90 ml/min per 1.73 m^2^ as normal, without separating hyperfiltration. Defining hyperfiltration periods was difficult because of the frequent fluctuations in creatinine measurements leading to oscillations between normal status and hyperfiltration. Finally, this study focused on the risk of AKI around CM exposure and did not evaluate the potential impact of these events on long-term kidney function.

### Generalizability

The study population consisted mainly of young adults, the majority with an SS genotype, preserved kidney function and limited comorbidity burden. Although age and reduced baseline eGFR were accounted for in the analysis, the small number of older patients or those with a baseline eGFR below 60 ml/min per 1.73 m^2^ suggests caution when extrapolating the results to these populations, especially as they were identified at risk for CA-AKI.^10^^,^^12^ Consistent with previous literature, increasing age was associated with a high RI of AKI in our cohort. The proportion of patients with confirmed albuminuria, a marker of SCD nephropathy and risk factor for CA-AKI,^43^ was consistent with published estimates of up to 27% in young adults with SCD.^44^

Hospitalized patients with SCD usually receive abundant hydration, including sodium bicarbonates, during hospitalization for vaso-occlusive crises, which may have acted as a nephroprotective factor around CM exposure, although the effectiveness of hydration in preventing AKI following CM exposure has not been demonstrated outside of specific clinical situations.^45^, ^46^, ^47^ It is therefore difficult to generalize our findings outside of this context, especially given our conservative definition of AKI, most cases in the full follow-up analysis also occurred during hospitalization. Lastly, the SCCS model does not allow for a formal assessment of the role of repeated exposures to CM, within a short time frame as a risk factor for AKI, or on the mid- or long-term as a risk factor for CKD. These remain to be explored by other study designs.

### Conclusion

In this cohort of hospitalized adults with SCD, which consisted mainly in young adults preserved kidney function and few comorbidities, the RI of AKI was twice as high both before and after CM exposure, suggesting that the observed association reflects the underlying clinical conditions and other related medical interventions, rather than a causal effect of CM itself.

## Disclosure

All the authors declared no competing interests.

## Data Sharing Statement

Raw data cannot be shared with non-GPUH staff without specific authorization from the GPUH CDW Scientific and ethics committee. R scripts are available at request to the corresponding author.

## Supplementary Material

Supplementary file (PDF)

**Figure S1**. Relative incidence of AKI according to age groups in the main analysis.

**Figure S2**. Gap time between AKI and the subsequent episode of AKI in the main analysis.

**Figure S3**. Gap time between AKI event and end of observation in years in the main analysis.

**Figure S4**. Relative incidence of AKI in the main analysis during the pre and postexposure period depending on the length of the pre-exposure period.

**Figure S5**. Delay between CM exposure and creatinine measurement in the main analysis.

**Table S1**. RECORD and STROBE checklists.

**Table S2**. Characteristics of patients included in the full follow-up analysis.

**Table S3**. Count of events and total duration of exposure periods for the full follow-up analysis.

**Table S4**. Relative incidence of AKI in the full follow-up analyses.

**Table S5**. Sensitivity analyses for both main analysis and full-follow up.
