# Supplementary material for: Contrast-Associated AKI in Hospitalized Adults With Sickle Cell Disease
Source: Kidney Int Rep. 2026 May 14;11(8):106600. doi: 10.1016/j.ekir.2026.106600 (PMC13264182; doi:10.1016/j.ekir.2026.106600)
Supplement: Supplementary file (PDF) [file mmc1.pdf]

Supplementary information

	Item No.	STROBE items	RECORD items	Location in manuscript where items are reported
	1	(a) Indicate the study's design with a commonly used term in the title or the abstract (b) Provide in the abstract an informative and balanced summary of what was done and what was found	RECORD 1.1: The type of data used should be specified in the title or abstract. When possible, the name of the databases used should be included.<br><br>RECORD 1.2: If applicable, the geographic region and timeframe within which the study took place should be reported in the title or abstract.<br><br>RECORD 1.3: If linkage between databases was conducted for the study, this should be clearly stated in the title or abstract.	Page 1 and 3 (abstract)
Background rationale	2	Explain the scientific background and rationale for the investigation being reported		Page 5-6 (introduction)
Objectives	3	State specific objectives, including any prespecified hypotheses		Page 6 (introduction)
Study Design	4	Present key elements of study design early in the paper		Page 7 (Materials and method: study design)
Setting	5	Describe the setting, locations, and relevant dates, including periods of recruitment, exposure, follow-up, and data collection		Page 7 (Materials and methods: study design)
Participants	6	*(a) Cohort study* - Give the eligibility criteria, and the sources and methods of selection of participants. Describe methods of follow-up<br><br>*Case-control study* - Give the eligibility criteria, and the sources and methods of case ascertainment and control	RECORD 6.1: The methods of study population selection (such as codes or algorithms used to identify subjects) should be listed in detail. If this is not possible, an explanation should be provided.	Page 7-8 (Materials and methods: inclusion criteria, data collection and identification of genotypes)

		selection. Give the rationale for the choice of cases and controls<br><br>*Cross-sectional study* - Give the eligibility criteria, and the sources and methods of selection of participants<br><br>*(b) Cohort study* - For matched studies, give matching criteria and number of exposed and unexposed<br><br>*Case-control study* - For matched studies, give matching criteria and the number of controls per case	RECORD 6.2: Any validation studies of the codes or algorithms used to select the population should be referenced. If validation was conducted for this study and not published elsewhere, detailed methods and results should be provided.<br><br>RECORD 6.3: If the study involved linkage of databases, consider use of a flow diagram or other graphical display to demonstrate the data linkage process, including the number of individuals with linked data at each stage.	
Variables	7	Clearly define all outcomes, exposures, predictors, potential confounders, and effect modifiers. Give diagnostic criteria, if applicable.	RECORD 7.1: A complete list of codes and algorithms used to classify exposures, outcomes, confounders, and effect modifiers should be provided. If these cannot be reported, an explanation should be provided.	Page 7-11 (Materials and methods: inclusion criteria, data collection and identification of genotypes, adjustment of time varying confounders, primary outcome)
Data sources/ measuremen t	8	For each variable of interest, give sources of data and details of methods of assessment (measurement).<br><br>Describe comparability of assessment methods if there is more than one group		Page 9-11 (Materials and methods: inclusion criteria, data collection and identification of genotypes, adjustment of time varying confounders, primary outcome)
Bias	9	Describe any efforts to address potential sources of bias		Page 8, 10-13 (Materials and methods: data collection and identification of genotypes, self-controlled case series, adjustment of time varying confounders, primary outcome, model assumptions)
Study size	10	Explain how the study size was arrived at		Page 8 8 (Materials and methods: inclusion criteria)
Quantitative variables	11	Explain how quantitative variables were handled in the analyses. If applicable, describe which groupings were chosen, and why		Page 7-11 (Materials and methods: inclusion criteria, data collection and identification of genotypes, adjustment of

				time varying confounders, primary outcome)
Statistical methods	12	(a) Describe all statistical methods, including those used to control for confounding<br><br>(b) Describe any methods used to examine subgroups and interactions<br><br>(c) Explain how missing data were addressed<br><br>(d) *Cohort study* - If applicable, explain how loss to follow-up was addressed<br><br>*Case-control study* - If applicable, explain how matching of cases and controls was addressed<br><br>*Cross-sectional study* - If applicable, describe analytical methods taking account of sampling strategy<br><br>(e) Describe any sensitivity analyses		Page 8-13, Figure 1<br><br>(Materials and methods: self-controlled case series, model assumptions)
Data access and cleaning methods		..	RECORD 12.1: Authors should describe the extent to which the investigators had access to the database population used to create the study population.<br><br>RECORD 12.2: Authors should provide information on the data cleaning methods used in the study.	Page 7<br><br>(Materials and methods: study design)
Linkage		..	RECORD 12.3: State whether the study included person-level, institutional-level, or other data linkage across two or more databases. The methods of linkage and methods of linkage quality evaluation should be provided.	/
Participants	13	(a) Report the numbers of individuals at each stage of the study (*e.g.*, numbers potentially eligible, examined for eligibility, confirmed eligible, included in the study, completing follow-up, and analysed)	RECORD 13.1: Describe in detail the selection of the persons included in the study (*i.e.,* study population selection) including filtering based on data quality, data availability and linkage. The selection of included	Page 15, Figure 2<br><br>(Results: study population)

		(b) Give reasons for non-participation at each stage.<br><br>(c) Consider use of a flow diagram	persons can be described in the text and/or by means of the study flow diagram.	
Descriptive data	14	(a) Give characteristics of study participants (*e.g.*, demographic, clinical, social) and information on exposures and potential confounders<br><br>(b) Indicate the number of participants with missing data for each variable of interest<br><br>(c) *Cohort study* - summarise follow-up time (*e.g.*, average and total amount)		Page 15, Table 1<br><br>(Results: study population)
Outcome data	15	*Cohort study* - Report numbers of outcome events or summary measures over time<br><br>*Case-control study* - Report numbers in each exposure category, or summary measures of exposure<br><br>*Cross-sectional study* - Report numbers of outcome events or summary measures		Page 15-16, Table 2, figure 3<br><br>(Results: study population, relative incidences)
Main results	16	(a) Give unadjusted estimates and, if applicable, confounder-adjusted estimates and their precision (e.g., 95% confidence interval). Make clear which confounders were adjusted for and why they were included<br><br>(b) Report category boundaries when continuous variables were categorized<br><br>(c) If relevant, consider translating estimates of relative risk into absolute risk for a meaningful time period		Page 16, Table 3<br><br>(Results: relative incidence)
Other analyses	17	Report other analyses done—e.g., analyses of subgroups and interactions, and sensitivity analyses		Page 16-17, Table S2-S5, Figure S1-S5<br><br>(Results: sensitivity analyses and verification of the model assumptions)
Key results	18	Summarise key results with reference to study objectives		Page 17

				(Discussion: key results)
Limitations	19	Discuss limitations of the study, taking into account sources of potential bias or imprecision. Discuss both direction and magnitude of any potential bias	RECORD 19.1: Discuss the implications of using data that were not created or collected to answer the specific research question(s). Include discussion of misclassification bias, unmeasured confounding, missing data, and changing eligibility over time, as they pertain to the study being reported.	Page 17-18<br><br>(Discussion: strength and limitations)
Interpretation	20	Give a cautious overall interpretation of results considering objectives, limitations, multiplicity of analyses, results from similar studies, and other relevant evidence		Page 17<br><br>(Discussion: interpretation)
Generalisability	21	Discuss the generalisability (external validity) of the study results		Page 19<br><br>(Discussion: Generalisability)
Funding	22	Give the source of funding and the role of the funders for the present study and, if applicable, for the original study on which the present article is based		No specific funding
Accessibility of protocol, raw data, and programming code		..	RECORD 22.1: Authors should provide information on how to access any supplemental information such as the study protocol, raw data, or programming code.	Page 20

Table S1: RECORD and STROBE checklist

**Variables**	**Full follow-up analysis**
Number of patients	541
Median age at inclusion	29.8 (21.9-41.0)
Women	300 (55.5 %)
Genotype	
SS	458 (84.7 %)
SC	48 (8.8 %)
Sβ+	11 (2.0 %)
Sβ0	11 (2.0 %)
Unclassified	13 (2.4 %)
Comorbidities before first AKI	
acute chest syndrome/pneumonia*	242 (44.7%)
hypertension	98 (18.1%)
heart failure	114 (21.1%)
ischemic heart disease	25 (4.6%)
rhythms and conductions disorders	49 (9.1%)
pulmonary embolism	68 (12.6%)
pulmonary arterial hypertension	41 (7.6%)
cerebrovascular accident	35 (6.5%)
diabetes mellitus	18 (3.3%)
malignancy	28 (5.2%)
Median observation length (years)	5.9 (4.0 – 7.1)
Total observation length (person-years)	2916.74
Baseline eGFR	
>= 90 mL/min/1.73m²	442 (81.7 %)
60-89 mL/min/1.73m²	52 (9.6 %)
45-59 mL/min/1.73m²	14 (2.6 %)
30 – 44 mL/min/1.73m²	21 (3.9 %)
15 – 29 mL/min/1.73m²	12 (2.2 %)

Patients with ≥3 outpatient urinary protein measurements	356 (65.8%)
Albuminuria identified in the first year of follow up	52 (14.6%)
Number of creatinine measurement	
Total	44 014
By patient	64 (35 – 110)
Number of outpatient urinary protein measurement	
Total	3517
By patient	7 (3 – 11)
Number of CM exposure	
Total	1888
By patient	2 (1-5)
Age at first CM exposure	30.4 (23.9-40.7)
Unexposed patients during the period of interest	74
Total number of imaging procedures with CM	2000
CT pulmonary angiogram	957 (47.9 %)
Abdomen and pelvis CT	300 (15.0 %)
CT of 3 or more regions	261 (13.1 %)
Chest CT	124 (6.2%)
Head CT	86 (4.3%)
Others	272 (13.6%)
Number of emergency department visits without subsequent hospitalization	
Total	2735
By patient	2 (0-5)
Number of hospitalizations without intensive care	
Total	8006
By patient	7 (3-16)

Number of hospitalizations with intensive care stay	
Total	756
By patient	0 (0-1)
Number of AKI	
Total	881
By patient	1 (1-2)
Age at first AKI	33.2 (25.3-44.0)
Number of stage 2 or 3 AKI	
Total	197
By patient	0 (0-1)
Number of stage 3 AKI	
Total	86
By patient	0 (0-0)

Table S2: characteristics of patients included in the full follow-up analysis. Categorical variables are presented with their count and percentage, numerical variables with the median, first and third quartile. AKI: acute kidney injury, CM: contrast media, CT: computed tomography, eGFR: estimated glomerular filtration rate. The total number of imaging procedures exceeds the total number of CM exposures because some patients underwent multiple imaging at the same time. *Acute chest syndrome and pneumonia are grouped together as no specific ICD-10 code exists for acute chest syndrome. Acute chest syndrome is often coded as pneumonia and pneumonia also fulfills its diagnostic criteria.

Exposure	Number of AKI	Person-years
**Total**	881	2916.74
**CM exposure**		
7 days pre-exposure	141	33.07
0-3 days post exposure	115	13.22
4-7 days post exposure	81	20.61
Outside 7 days pre- or postexposure	544	2849.84
**Settings of care**		
Emergency department visit without subsequent hospitalization	33	14.18
Hospitalization without intensive care stay	564	151.88
Hospitalization with intensive care stay	191	36.85
Outpatient care only	93	2713.82

Table S3: count of events and total duration of exposure periods in person-years for the full follow-up analysis.

Exposure	Relative incidence	95% CI	p
**Acute Kidney Injury (n= 881)**			
7 days pre CM exposure*	2.90	2.34-3.60	< 0.001
0-3 days post CM exposure*	3.11	2.48-3.90	< 0.001
4-7 days post CM exposure*	2.00	1.55-2.58	< 0.001
Emergency department visit without subsequent hospitalization**	4.60	3.17– 6.66	< 0.001
Hospitalization without intensive care stay**	96.64	76.80-121.60	< 0.001
Hospitalization with intensive care stay**	166.96	122.77-227.05	< 0.001
Terminal risk period***	4.51	3.38-6.03	< 0.001
**Stage 2 or 3 Acute Kidney Injury (n= 197)**			
7 days pre CM exposure*	4.69	3.00-7.32	< 0.001
0-3 days post CM exposure*	4.38	2.68-7.17	< 0.001
4-7 days post CM exposure*	4.43	2.75-7.11	< 0.001
Emergency department visit without subsequent hospitalization**	4.47	2.00-9.99	< 0.001
Hospitalization without intensive care stay**	92.57	55.00-155.80	< 0.001
Hospitalization with intensive care stay**	135.07	68.61-265.90	< 0.001
Terminal risk period***	9.40	5.29-16.71	< 0.001
**Stage 3 Acute Kidney Injury (n=86)**			
7 days pre CM exposure*	7.43	3.83-14.43	< 0.001
0-3 days post CM exposure*	7.99	3.87-16.51	< 0.001
4-7 days post CM exposure*	5.16	2.34-11.38	< 0.001
Emergency department visit without subsequent hospitalization**	8.63	3.04-24.49	< 0.001

Hospitalization without intensive care stay**	43.17	20.73-89.88	< 0.001
Hospitalization with intensive care stay**	113.90	37.17-349.03	< 0.001
Terminal risk period***	7.42	3.35-16.44	< 0.001

Table S4: Relative incidence of AKI in the full follow-up analyses. Analyses account for pre and post CM exposure, settings of care, CKD staging, age and a terminal risk period of 30 days. Analyses were not adjusted on CKD staging for stage 3 AKI given the lower number of events. *Reference is the period outside the 7 days before and 7 days after CM exposure for the same patient. **Reference is ambulatory care. ***Reference corresponds to the remainder of the observation period, excluding these 30 days.

	Main analyses (hospitalization-only)			Full follow-up analyses		
**Exposure**	**Relative incidence**	**95% CI**	**p**	**Relative incidence**	**95% CI**	**p**
**Acute Kidney Injury (analyses without terminal risk period)**						
7 days pre CM exposure*	2.54	2.03-3.17	< 0.001	2.95	2.38-3.66	< 0.001
0-3 days post CM exposure*	2.53	2.00-3.21	< 0.001	3.18	2.54-3.98	< 0.001
4-7 days post CM exposure*	1.58	1.21-2.07	< 0.001	2.02	1.56-2.61	< 0.001
Emergency department visit without subsequent hospitalization**	-	-	-	4.60	3.18 –6.66	< 0.001
Hospitalization without intensive care stay**	-	-	-	97.21	77.19-122.43	< 0.001
Hospitalization with intensive care stay**	1.64	1.28-2.12	< 0.001	161.73	119.00-219.81	< 0.001
**Acute Kidney Injury (First events only).**						
7 days pre CM exposure*	2.73	2.04-3.64	< 0.001	3.33	2.52-4.39	< 0.001
0-3 days post CM exposure*	2.75	2.04-3.71	< 0.001	3.68	2.77-4.87	< 0.001
4-7 days post CM exposure*	1.65	1.17-2.32	0.005	2.17	1.57-3.02	< 0.001
Emergency department visit without subsequent hospitalization**	-	-	-	5.39	3.36-8.65	< 0.001
Hospitalization without intensive care stay**	-	-	-	122.92	91.35-165.39	< 0.001
Hospitalization with intensive care stay**	1.85	1.33-2.58	< 0.001	221.97	148.57-331.64	< 0.001
Terminal risk period***	1.30	0.71-2.36	0.39	3.96	2.65-5.93	< 0.001
**Acute kidney injury with 2 pre CM exposure periods**						
7-4 days pre CM exposure*	2.85	1.97-4.13	< 0.001	4.62	3.35-6.35	< 0.001

3-0 days pre CM exposure*	2.63	1.87-3.70	< 0.001	4.77	3.57-6.37	< 0.001
0-3 days post CM exposure*	3.10	2.36-4.09	< 0.001	5.19	4.05-6.64	< 0.001
4-7 days post CM exposure*	1.74	1.27-2.40	< 0.001	3.23	2.45-4.28	< 0.001
Emergency department visit without subsequent hospitalization**	-	-	-	6.26	4.13-9.50	< 0.001
Hospitalization without intensive care stay**	-	-	-	41.83	35.03-49.96	< 0.001
Hospitalization with intensive care stay**	1.79	1.32-2.43	0.005	67.97	50.66-88.80	< 0.001
Terminal risk period***	1.86	1.14-3.05	0.01	4.60	3.46-6.11	< 0.001
**Acute Kidney Injury with sKDIGO definition.**						
7 days pre CM exposure*	2.44	2.06-3.68	< 0.001	2.82	2.26-3.50	< 0.001
0-3 days post CM exposure*	2.45	1.93-3.12	< 0.001	3.04	2.41-3.82	< 0.001
4-7 days post CM exposure*	1.53	1.17-2.01	0.002	1.94	1.50-2.53	< 0.001
Emergency department visit without subsequent hospitalization**	-	-	-	4.22	2.86-6.21	< 0.001
Hospitalization without intensive care stay**	-	-	-	103.16	81.43-130.68	< 0.001
Hospitalization with intensive care stay**	1.70	1.32-2.19	< 0.001	181.69	132.77-248.63	< 0.001
Terminal risk period***	1.80	1.19-2.68	0.005	4.56	3.40-6.12	< 0.001

Table S5 : various sensitivity analysis to investigate both main analyses and full follow-up analyses. Analyses account for pre and post CM exposure, settings of care, CKD staging, age and a terminal risk period of 30 days. Analyses were not adjusted on CKD staging for stage 3 AKI given the lower number of events. *Reference is the period outside the 7 days before and 7 days after CM exposure for the same patient. **Reference is hospitalization without intensive care stay for the main analyses and ambulatory care for the full follow-up analyses. ***Reference corresponds to the remainder of the observation period, excluding these 30 days.

	Item No.	Recommendation	Page No.
**Title and abstract**	1	(*a*) Indicate the study's design with a commonly used term in the title or the abstract	1, 3
		(*b*) Provide in the abstract an informative and balanced summary of what was done and what was found	3
**Introduction**			
Background/rationale	2	Explain the scientific background and rationale for the investigation being reported	5-6
Objectives	3	State specific objectives, including any prespecified hypotheses	6
**Methods**			
Study design	4	Present key elements of study design early in the paper	7 (study design)
Setting	5	Describe the setting, locations, and relevant dates, including periods of recruitment, exposure, follow-up, and data collection	7 (study design)
Participants	6	(*a*) *Cohort study*—Give the eligibility criteria, and the sources and methods of selection of participants. Describe methods of follow-up<br><br>*Case-control study*—Give the eligibility criteria, and the sources and methods of case ascertainment and control selection. Give the rationale for the choice of cases and controls<br><br>*Cross-sectional study*—Give the eligibility criteria, and the sources and methods of selection of participants	7-8 (inclusion criteria)
		(*b*) *Cohort study*—For matched studies, give matching criteria and number of exposed and unexposed<br><br>*Case-control study*—For matched studies, give matching criteria and the number of controls per case	

Variables	7	Clearly define all outcomes, exposures, predictors, potential confounders, and effect modifiers. Give diagnostic criteria, if applicable	11-12<br><br>(data collection and identification of genotypes, adjustment of time varying confounders, primary outcome)
Data sources/ measurement	8*	For each variable of interest, give sources of data and details of methods of assessment (measurement). Describe comparability of assessment methods if there is more than one group	8-13<br><br>(adjustment of time varying confounders, primary outcome)
Bias	9	Describe any efforts to address potential sources of bias	13, 14 (model assumptions)
Study size	10	Explain how the study size was arrived at	4 (study design)
Quantitative variables	11	Explain how quantitative variables were handled in the analyses. If applicable, describe which groupings were chosen and why	5,6 (data collection and identification of genotypes)
Statistical methods	12	(*a*) Describe all statistical methods, including those used to control for confounding	8-9, 13, figure 1 (self-controlled case series, adjustment of time varying confounders, model assumptions)
		(*b*) Describe any methods used to examine subgroups and interactions	/
		(*c*) Explain how missing data were addressed	/
		(*d*) *Cohort study*—If applicable, explain how loss to follow-up was addressed<br><br>*Case-control study*—If applicable, explain how matching of cases and controls was addressed<br><br>*Cross-sectional study*—If applicable, describe analytical methods taking account of sampling strategy	/
		(*e*) Describe any sensitivity analyses	13, 14 (model assumptions)
**Results**			

Participants	13*	(a) Report numbers of individuals at each stage of study—eg numbers potentially eligible, examined for eligibility, confirmed eligible, included in the study, completing follow-up, and analysed	15, 16, figure 2<br><br>(Study population)
		(b) Give reasons for non-participation at each stage	15, figure 2
		(c) Consider use of a flow diagram	figure 2
Descriptive data	14*	(a) Give characteristics of study participants (eg demographic, clinical, social) and information on exposures and potential confounders	15, 16-, table 1<br><br>(study population)
		(b) Indicate number of participants with missing data for each variable of interest	15, 16,table 1
		(c) *Cohort study*—Summarise follow-up time (eg, average and total amount)	15, table 1
Outcome data	15*	*Cohort study*—Report numbers of outcome events or summary measures over time	15, 16, table 2 and 3<br><br>(study population, relative incidences)
		Case-control study—Report numbers in each exposure category, or summary measures of exposure	
		Cross-sectional study—Report numbers of outcome events or summary measures	
Main results	16	(*a*) Give unadjusted estimates and, if applicable, confounder-adjusted estimates and their precision (eg, 95% confidence interval). Make clear which confounders were adjusted for and why they were included	16, table 2 and 3<br><br>(relative incidences)
		(*b*) Report category boundaries when continuous variables were categorized	/
		(*c*) If relevant, consider translating estimates of relative risk into absolute risk for a meaningful time period	/

Other analyses	17	Report other analyses done—eg analyses of subgroups and interactions, and sensitivity analyses	16, 17, table S1 to S5<br><br>(sensitivity analyses and verification of the model assumption)
**Discussion**			
Key results	18	Summarise key results with reference to study objectives	18 (key results)
Limitations	19	Discuss limitations of the study, taking into account sources of potential bias or imprecision. Discuss both direction and magnitude of any potential bias	18-20 (strengths and limitations)
Interpretation	20	Give a cautious overall interpretation of results considering objectives, limitations, multiplicity of analyses, results from similar studies, and other relevant evidence	18-20 (interpretation)
Generalisability	21	Discuss the generalisability (external validity) of the study results	20 (generalizability)
**Other information**			
Funding	22	Give the source of funding and the role of the funders for the present study and, if applicable, for the original study on which the present article is based	/

STROBE Statement checklist .

Figure legends

Figure S1: Relative incidence of AKI according to the age group in the main analyses. Dashed lines represent the 95% confidence interval.

Figure S2: Gap time between AKI and the subsequent episode of AKI in the main analysis. Events separated by 7 days or more were considered as different events.

Figure S3: Gap time between AKI event and end of observation in years in the main analysis.

Figure S4: Relative incidence of AKI in the main analysis during the pre-exposure period (left) and post exposure period of 0-3 days (right) and their 95% CI, depending on the length of the pre-exposure period (from 0 to 21 days during the same hospitalization).

Figure S5: Delay between CM exposure and creatinine measurement in the main analysis.

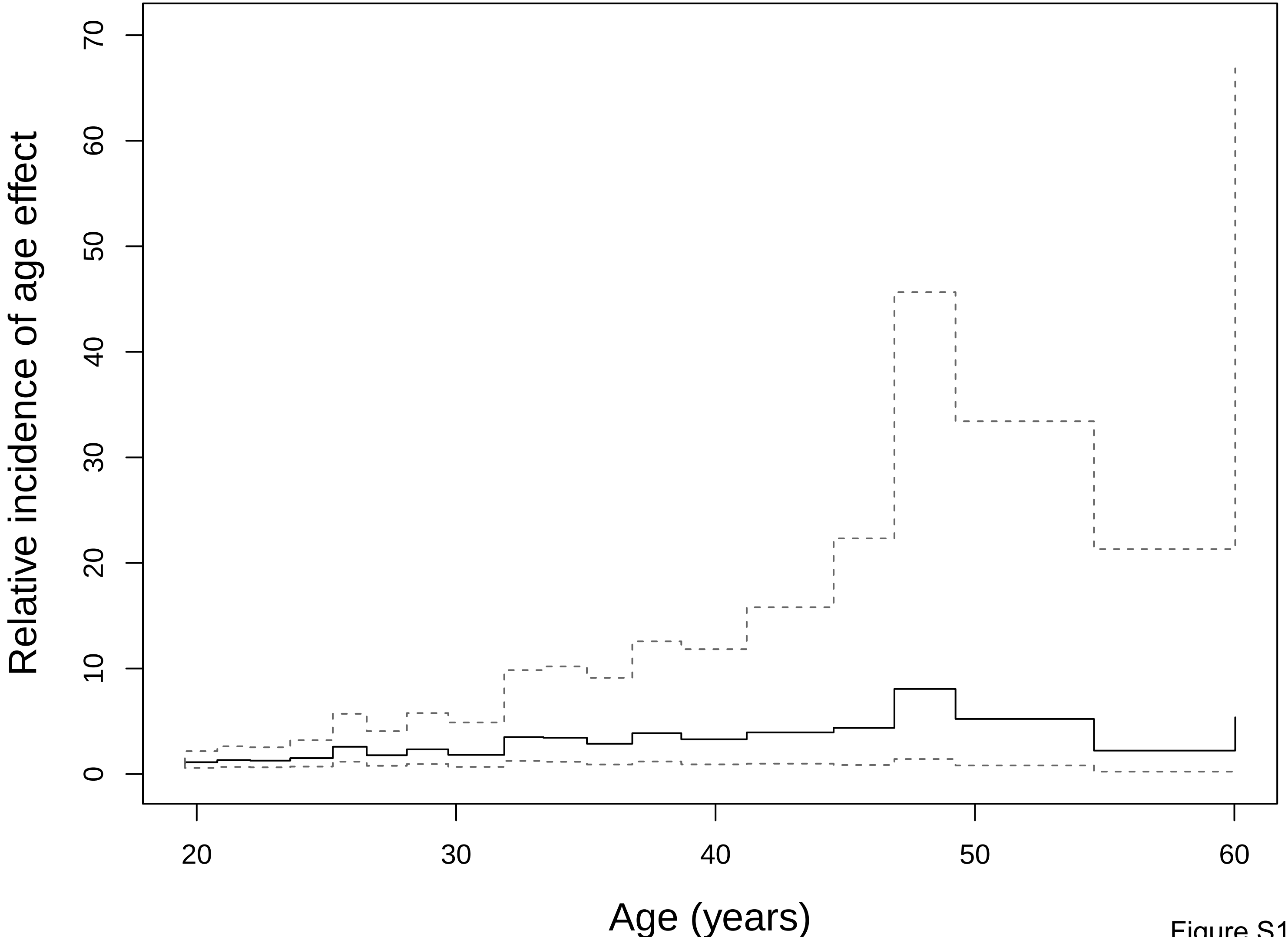

Relative incidence of age effect
0
10
20
30
40
50
60
70
20
30
40
50
60
Age (years)

Figure S1

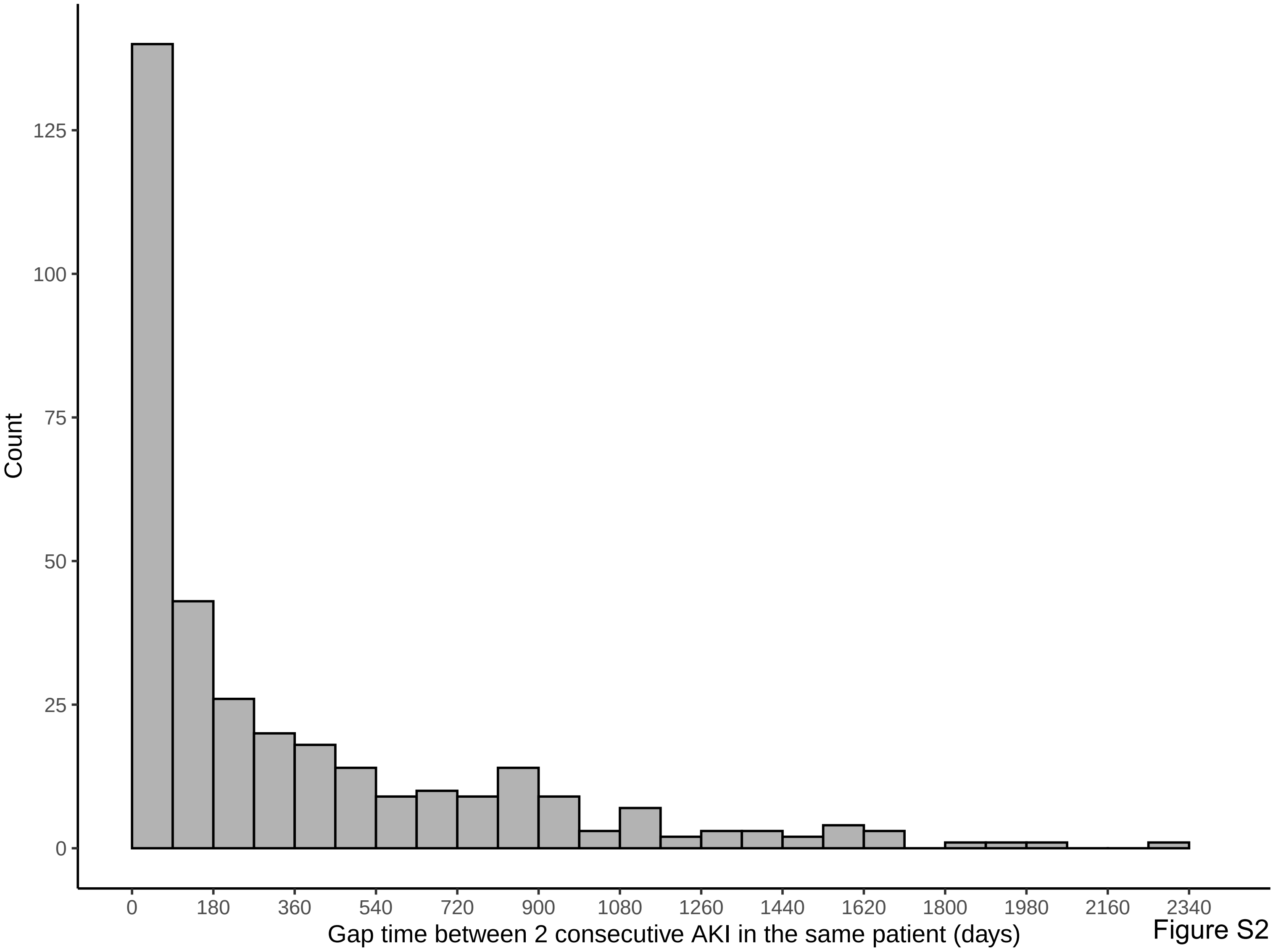

Count
125
100
75
50
25
0
0
180
360
540
720
900
1080
1260
1440
1620
1800
1980
2160
2340
Gap time between 2 consecutive AKI in the same patient (days)

Figure S2

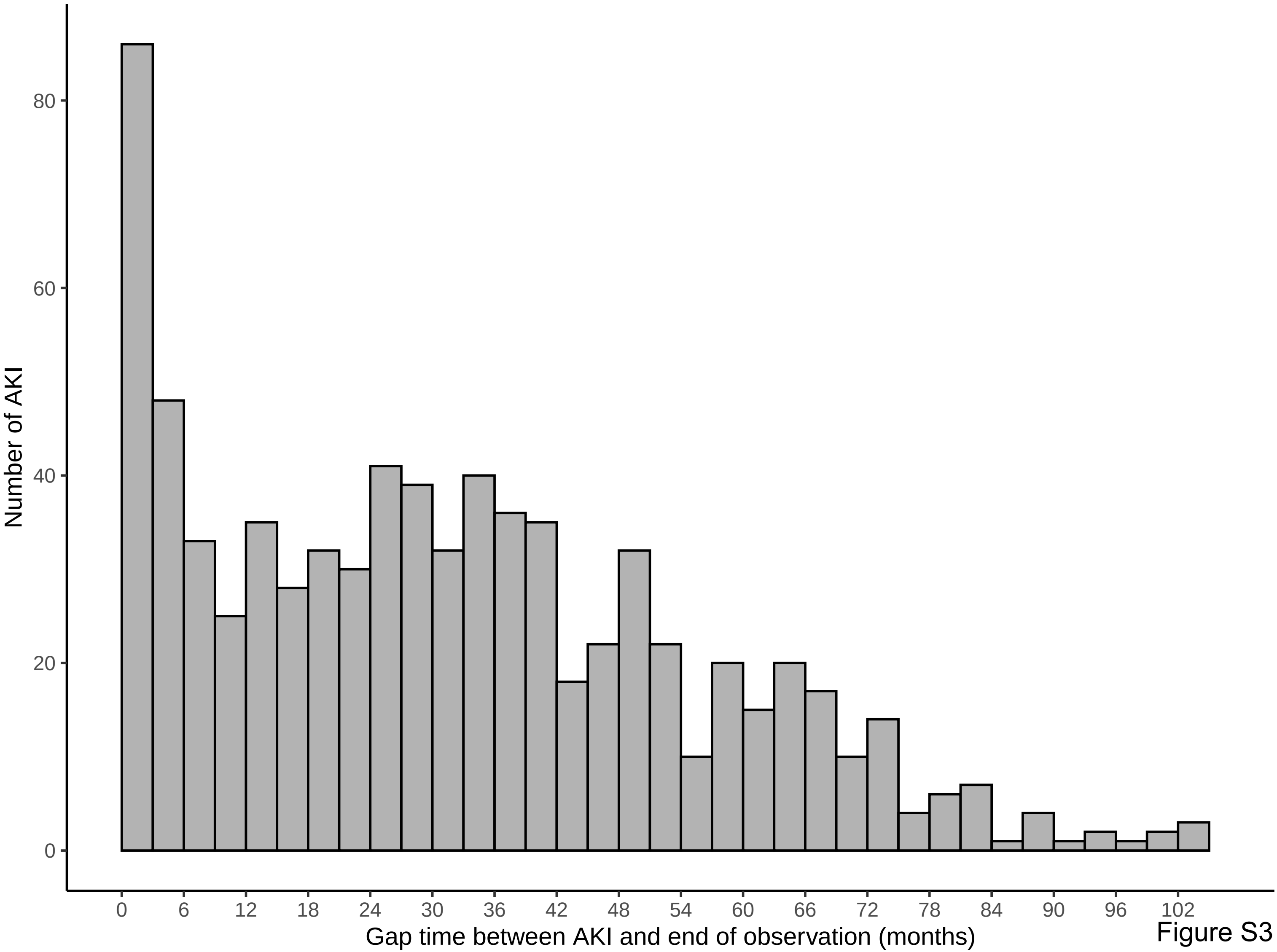
Number of AKI
80
60
40
20
0
0
6
12
18
24
30
36
42
48
54
60
66
72
78
84
90
96
102
Gap time between AKI and end of observation (months)
Figure S3

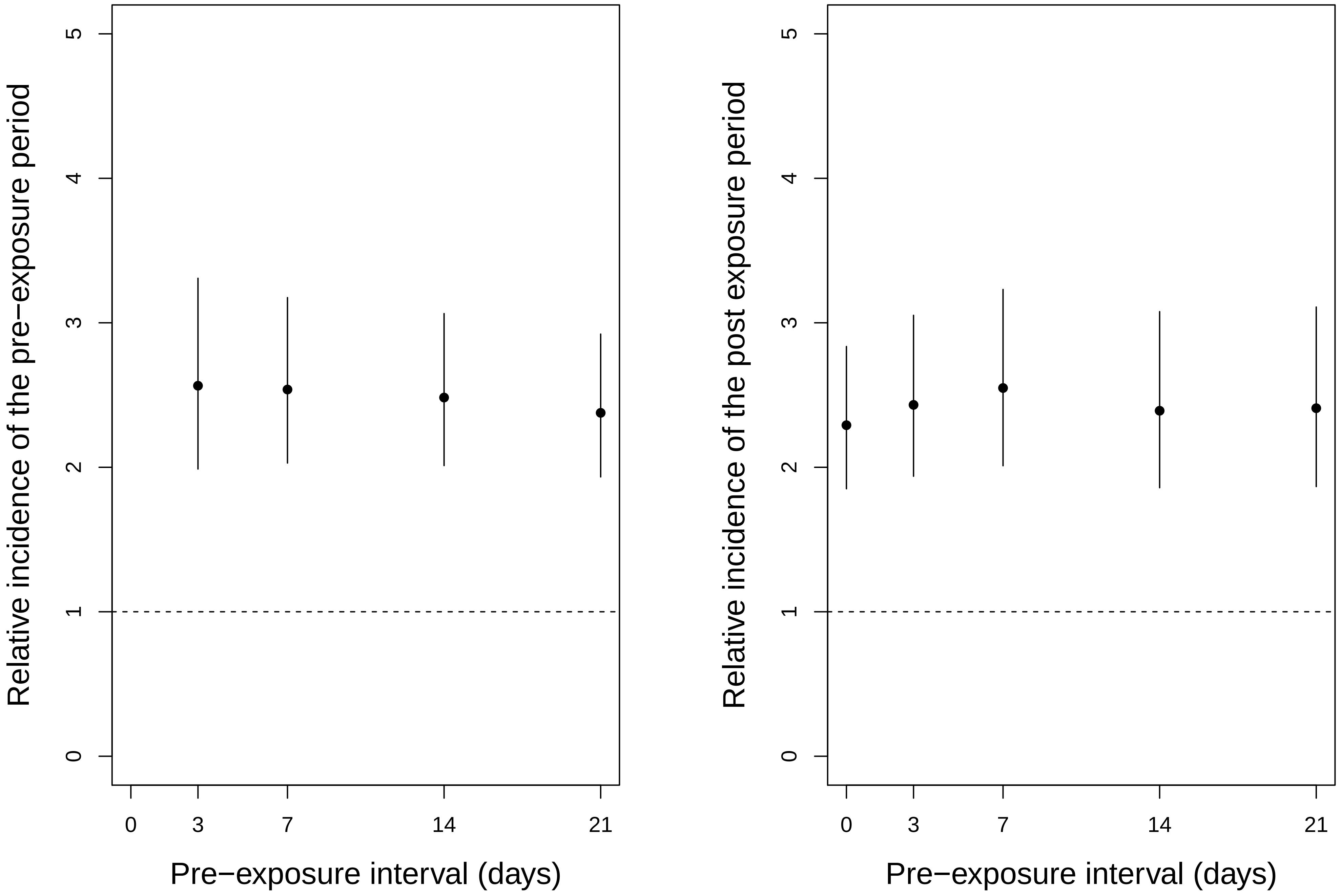

Relative incidence of the pre−exposure period
Pre−exposure interval (days)
0
1
2
3
4
5
0
3
7
14
21
Relative incidence of the post exposure period
Pre−exposure interval (days)
0
1
2
3
4
5
0
3
7
14
21

Figure S4

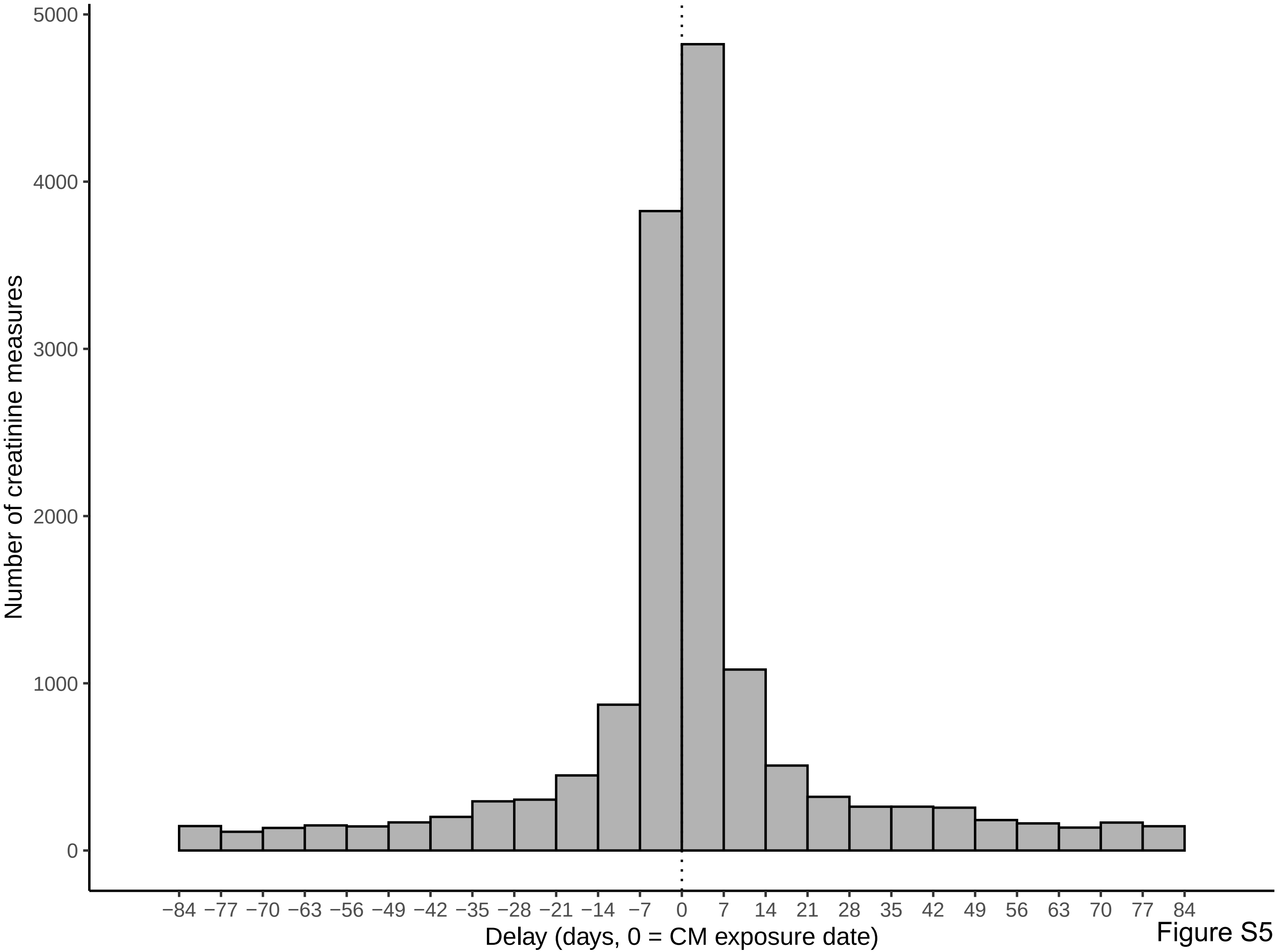

Number of creatinine measures
5000
4000
3000
2000
1000
0
−84 −77 −70 −63 −56 −49 −42 −35 −28 −21 −14 −7 0 7 14 21 28 35 42 49 56 63 70 77 84
Delay (days, 0 = CM exposure date)

Figure S5
